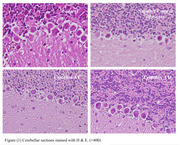# Neuroprotective Effect of Urolithin A against Cerebellum Changes in Streptozotocin‐Induced Alzheimer’s Disease Rat Model

**DOI:** 10.1002/alz.087882

**Published:** 2025-01-09

**Authors:** Mona Taghizade Salari, Leila Khani, Morteza Behnam‐Rassouli, Kianoush Gholami, Hananeh Ahmadnia, Maede Hasanpour, Milad Iranshahy, Mehrdad Iranshahi

**Affiliations:** ^1^ Ferdowsi University of Mashhad, Mashhad, Razavi Khorasan Iran (Islamic Republic of); ^2^ Mashhad University of Medical Sciences, Mashhad, Razavi Khorasan Iran (Islamic Republic of); ^3^ Tehran University of Medical Sciences, Tehran, Tehran Iran (Islamic Republic of); ^4^ Wilfrid Laurier University, Waterloo, ON Canada

## Abstract

**Background:**

Microbiota of the distal part of the intestine produces Urolithin A (Uro A) as a derivative of ellagitannins hydrolysis. Recently, the mitophagy, anti‐inflammatory, and antioxidant properties of Uro A have focused more attention on its probable beneficial effects on neurodegenerative states. The purpose of this research was to study the impact of Uro A on the histopathology of the cerebellum in a rat model of streptozotocin‐induced Alzheimer’s disease.

**Methods:**

Young male Wistar rats underwent stereotaxic surgery and infused streptozotocin (STZ; 3 mg/Kg body weight, dissolved in 10 µl vehicle of ascorbic acid‐saline 0.1%), intracerebroventricularly. After that, rats of experimental groups 1 and 2 were administered daily (i.p.) Uro A at two different doses; 10 and 20 mg/kg body weight, respectively. At the end of the experimental period (2 weeks), rats were deeply anesthetized, perfused (10% formalin solution), and decapitated, and their brains were removed and post‐fixed in the same fixator. Then, the cerebellums were separated and processed for histological preparation. Paraffin sections (5 µm thickness) were stained (H&E) and examined under a light microscope.

**Result:**

The microscopic photos of cerebellum sections of control, negative control, and experimental rats are presented in Figure 1. The photos show that, in comparison with the control, the disruption in the continuity of the Purkinje cell layer, as well as the abnormal morphology and lower density of Purkinje cells are obvious in the negative control section.

**Conclusion:**

It seems that Uro A treatment could somehow limit the destructive effects of STZ on the cerebellar Purkinje cell layer. Previously, decreased densities of Purkinje cells and notable morphological changes have been reported in the cerebellum of Alzheimer’s patients (Baloyannis et al., 2000).